# Evaluation of 12 GWAS-drawn SNPs as biomarkers of rheumatoid arthritis response to TNF inhibitors. A potential SNP association with response to etanercept

**DOI:** 10.1371/journal.pone.0213073

**Published:** 2019-02-28

**Authors:** Aida Ferreiro-Iglesias, Ariana Montes, Eva Perez-Pampin, Juan D. Cañete, Enrique Raya, Cesar Magro-Checa, Yiannis Vasilopoulos, Rafael Caliz, Miguel Angel Ferrer, Beatriz Joven, Patricia Carreira, Alejandro Balsa, Dora Pascual-Salcedo, Francisco J. Blanco, Manuel J. Moreno-Ramos, Sara Manrique-Arija, María del Carmen Ordoñez, Juan Jose Alegre-Sancho, Javier Narvaez, Federico Navarro-Sarabia, Virginia Moreira, Lara Valor, Rosa Garcia-Portales, Ana Marquez, Juan J. Gomez-Reino, Javier Martin, Antonio Gonzalez

**Affiliations:** 1 Experimental and Observational Rheumatology and Rheumatology Unit, Instituto de Investigacion Sanitaria—Hospital Clinico Universitario de Santiago, Santiago de Compostela, Spain; 2 Arthritis Unit, Rheumatology Dpt, Hospital Clinic and IDIBAPS, Barcelona, Spain; 3 Department of Rheumatology, Hospital Clínico San Cecilio, Granada, Spain; 4 Department of Rheumatology, Leiden University Medical Center, Leiden, The Netherlands; 5 Department of Biochemistry and Biotechnology, University of Thessaly, Larissa, Greece; 6 Rheumatology Unit, Hospital Universitario Virgen de las Nieves, Granada, Spain; 7 Reumatology Department, Hospital 12 de Octubre, Madrid, Spain; 8 Department of Rheumatology, Instituto de Investigación Hospital Universitario La Paz, Hospital Universitario La Paz, Madrid, Spain; 9 Department of Immunology, Instituto de Investigación Hospital Universitario La Paz, Madrid, Spain; 10 Rheumatology Department, Instituto de Investigacion Biomedica–Complejo Hospitalario Universitario A Coruna, A Coruna, Spain; 11 Department of Rheumatology, Hospital Virgen de la Arrixaca, Murcia, Spain; 12 Servicio de Reumatología, HRU Carlos Haya, Universidad de Málaga, Instituto de Investigación Biomédica de Málaga (IBIMA), Málaga, Spain; 13 Department of Rheumatology, Hospital Doctor Peset, Valencia, Spain; 14 Department of Rheumatology, Hospital Universitario de Bellvitge, Barcelona, Spain; 15 Rheumatology Unit, Hospital Universitario Virgen Macarena, Sevilla, Spain; 16 Rheumatology Unit, Hospital General Universitario Gregorio Marañón, Madrid, Spain; 17 Department of Rheumatology, Hospital Virgen de la Victoria, Málaga, Spain; 18 Instituto de Parasitología y Biomedicina López-Neyra, CSIC, Granada, Spain; Memorial University of Newfoundland, CANADA

## Abstract

Research in rheumatoid arthritis (RA) is increasingly focused on the discovery of biomarkers that could enable personalized treatments. The genetic biomarkers associated with the response to TNF inhibitors (TNFi) are among the most studied. They include 12 SNPs exhibiting promising results in the three largest genome-wide association studies (GWAS). However, they still require further validation. With this aim, we assessed their association with response to TNFi in a replication study, and a meta-analysis summarizing all non-redundant data. The replication involved 755 patients with RA that were treated for the first time with a biologic drug, which was either infliximab (n = 397), etanercept (n = 155) or adalimumab (n = 203). Their DNA samples were successfully genotyped with a single-base extension multiplex method. Lamentably, none of the 12 SNPs was associated with response to the TNFi in the replication study (p > 0.05). However, a drug-stratified exploratory analysis revealed a significant association of the *NUBPL* rs2378945 SNP with a poor response to etanercept (B = -0.50, 95% CI = -0.82, -0.17, p = 0.003). In addition, the meta-analysis reinforced the previous association of three SNPs: rs2378945, rs12142623, and rs4651370. In contrast, five of the remaining SNPs were less associated than before, and the other four SNPs were no longer associated with the response to treatment. In summary, our results highlight the complexity of the pharmacogenetics of TNFi in RA showing that it could involve a drug-specific component and clarifying the status of the 12 GWAS-drawn SNPs.

## Introduction

Rheumatoid arthritis (RA) is a systemic autoimmune disease that until the late 1990s led to permanent disability, low life quality and increased mortality [1]. The development of targeted drugs, pioneered by TNF inhibitors (TNFi), transformed this poor clinical evolution. Now, it is possible to obtain long-term clinical remission or low disease activity in an important proportion of patients [1,2]. The remaining patients (about 30%) will not appropriately respond to a specific drug although they may respond to another. Therefore, biomarkers for prediction of the response will improve the benefits and avoid the unnecessary costs and side effects of the targeted drugs [3,4].

The goal of predicting the response to treatment in RA patients has been pursued in many research areas [3,4]. One of these areas has been genetics, where candidate-gene and genome-wide studies (GWAS) have been performed [5,6]. They have been primarily concentrated on the response to three TNFi: infliximab, adalimumab, and etanercept, as the most widely used biologic Disease Modifying Anti-Rheumatic Drug (bDMARD). The initial studies were focused on candidate genes, with many addressing the TNFα gene [7,8]. These studies were small, probably expecting polymorphisms with an important influence in the drug effect [6,9]. Unfortunately, their findings were not reproducible showing the initial expectations were too optimistic [6,8,10–12]. More recently, several large studies have been reported including many hundreds or thousands of RA patients [12–17]. They have demonstrated promising SNPs that are associated with the response to TNFi at various levels of evidence. Some appeared in candidate-gene studies, as the *PTPRC* rs10919563 SNP, which approached the GWAS-level of significance combining three large studies [15–17]. Others have been highlighted in GWAS [11–14,18,19], like the four SNPs we attempted to validate in a previous work [20], and the 12 SNPs that we have selected now.

We have drawn these 12 SNPs from the three largest published GWAS [12–14]. Two of them included the same ≈ 2700 patients that were analyzed according to different protocols [12,14], while the third GWAS counted with 1278 patients [13]. The 12 SNPs fulfilled the requirements of replicability established on the respective GWAS, although none of them reached the GWAS-level of significance (p < 5 x10^-8^). Nevertheless, the *CD84* rs6427528 was associated with p = 8 x10^-8^, but only with the response to etanercept, not with the response to infliximab or adalimumab [14]. This result signaled the possibility of drug-specific biomarkers within the response to the TNFi. Indeed, other studies have shown drug-specific genetic [19,21–23] and protein biomarkers [24]. This specificity could be consequence of the known differences in structure, pharmacokinetics and interactions between the three TNFi [25,26]. Therefore, we have addressed the replication of the 12 SNPs considering the three TNFi together and separately. In addition, we have completed the SNPs assessment by meta-analysis to combine our results with the data from previous studies.

## Material and methods

### Patients

A total of 788 patients with RA according to the American College of Rheumatology classification criteria [27] were included. They were either of self-reported Spanish European ancestry (n = 731) recruited in 15 Spanish Rheumatology Units, or of Greek European ancestry recruited in two Greek hospitals (n = 57). All provided blood samples for DNA extraction and their informed written consent. The study was conducted according to the principles of the Declaration of Helsinki (2013) and was approved by the Comité Ético de Investigación Clínica del Hospital Clínic de Barcelona, the Comité Ético de Investigación Clínica de Centro de Granada, University of Thessaly and Medical School Ethics Committee, the Comité Ético de Investigación Clínica del Hospital 12 de Octubre, the Comité de Ética de la Investigación con Medicamentos del Hospital La Paz, the Comité Ético de Investigación del Hospital Virgen de la Arrixaca, the Comité de Ética de la Investigación del Hospital Regional Universitario Carlos Haya, the Comité Ético de Investigación Clínica del Hospital Universitario Doctor Peset, the Comitè d'Ètica d’Investigació Clínica de l’Hospital Universitari de Bellvitge, the Comité de Ética de la Investigación de los Hospitales Universitarios Virgen Macarena-Virgen del Rocío, the Comité de Ética de la Investigación con Medicamentos del Hospital Universitario Gregorio Marañón, the Comité de Ética de la Investigación Málaga Noroeste, the Comité de Bioética del Consejo Superior de Investigaciones Científica and by the Comite Etico de Investigacion Clinica de Galicia (Santiago de Compostela, Spain). All the patients were treated with a TNFi as the first bDMARD between 2000 and 2011. The indication of treatment, the choice of drug, and the control of clinical evolution were performed with independence of this study during the standard care of the patients. Evaluations included Disease Activity Score 28 (DAS28) at the start of treatment and at 3, 6, and 12 months. DAS28 is a composite index of RA activity including the number of tender joints and swollen joints (28 joints maximum), erythrocyte sedimentation rate, and global patient health status assessment [28]. Patients with baseline DAS28 < 3.2 (i.e. showing low activity, n = 13), and samples failing most genotypes (n = 20) were excluded from further analysis. The remaining 755 patients were distributed as follows: 397 treated with infliximab, 155 treated with etanercept, and 203 treated with adalimumab. Their clinical characteristics are detailed in Table 1. We have sufficient information to analyze response to treatment of 452 patients at 3 months, 689 patients at 6 months and 531 at 12 months. The corresponding raw data are provided in Supporting information S1 Table.

**Table 1:** Characteristics of the 755 patients with RA included in the study.

Characteristic	Value
Female N (%)	624 (82.7)
Age at diagnosis, mean ± SD years	43.2 ± 14.1
Diagnosis to TNFi treatment, mean ± SD years	7.9 ± 7.5
Rheumatoid factor positive, N (%)	560 (74.4)
Anti-CCP positive, N (%)*	423 (70.6)
Erosive arthritis. N (%)*	422 (70.7)
Ever smokers, N (%)*	100 (20.0)
DAS28 at baseline, mean ± SD	5.8 ± 1.1
HAQ at baseline, mean ± SD*	1.5 ± 0.7
Concomitant DMARDs, N (%)	547 (95.0)
TNFi drug, N (%)	
Infliximab	397 (52.6)
Etanercept	155 (20.5)
Adalimumab	203 (26.9)
EULAR response, N (%)	
3 months (N = 452)	
good	137 (30.3)
moderate	221 (48.9)
non responder	94 (20.8)
6 months (N = 689)	
good	262 (38.0)
moderate	291 (42.2)
non responder	136 (19.7)
12 months (N = 531)	
good	242 (45.6)
moderate	193 (36.4)
non responder	96 (18.1)

* Data was available from <85% of the patients: 599 for anti-CCP antibodies, 597 for erosive arthritis, 501 for smoking, 528 for baseline HAQ and 576 for concomitant DMARDs.

Abbreviations: DMARD = disease-modifying anti-rheumatic drug; SD = standard deviation; DAS28 = Disease Activity Score 28 joints; HAQ = Health Assessment Questionnaire; EULAR = The European League Against Rheumatism.

### Genotyping assays

Twelve SNPs (Table 2) were selected because of their reported association with response to TNFi in published GWAs [12–14]. These SNPs were genotyped with a multiplex single-base extension technology (SNaPshot Multiplex Kit from Applied Biosystems, Foster City, CA) starting with DNA amplification in a multiplex PCR reaction (KAPA2G fast HotStart, Kapa Biosystems, Woburn MA). Ten per cent of the samples were re-genotyped for quality control. Primers and probes used for these analyses are available in Supporting Information S2 Table.

**Table 2:** SNPs associated with response to TNFi in RA GWAS selected for this study.

Discovery	SNP	Location	p
Plant *et al*. ^a^	rs7962316	*BC118985/BTP1*	0.02
	rs1350948	*Chr11*: *23518405*	0.008
	rs4694890	*TEC*	0.006
Umicevic Mirkov *et al*. ^a^	rs2378945	*NUBPL*	0.0007
	rs12142623	*Chr1*: *185557029*	0.0002
	rs1568885	*Chr7*: *13604056*	0.00017
	rs1447722	*Chr3*: *141037143*	0.00016
	rs1813443	*CNTN5*	0.00014
	rs4651370	*Chr1*:*185505715*	0.00011
	rs4411591	*LOC100130480*	0.00005
	rs7767069	*Chr6*: *68827284*	0.00008
Cui *et al*. ^a^	rs6427528	*CD84*	0.00000008 ^b^

^a^ The three GWAS used ΔDAS28 as outcome, including 1278, 2703 and 2706 partly overlapping sets of RA patients, respectively. Assessment was done at 6 months, 14 weeks and at any time between 3 and 12 months, respectively.

^b^ Only in the 733 RA patients treated with etanercept.

### Statistical analyses

We have used reproducibility of genotypes, genotype call rate, the Hardy-Weinberg equilibrium (HWE) and coincidence with SNP frequencies in the HapMap Toscani in Italy (TSI) collection [29] as quality control measures. The response to TNFi was assessed primarily as change in DAS28 (ΔDAS28 = DAS28 _baseline_−DAS28_follow-up_) at 6 months of follow-up. Additionally, we have also considered ΔDAS28 at the 3 and 12 month points and the responder (good + moderate) versus non-responder classification according to the European League Against Rheumatism (EULAR) criteria [30]. The EULAR criteria divide patients into three classes based on change in DAS28 from baseline (ΔDAS28) and DAS28 at the time of evaluation: good responders are those with ΔDAS28 ≥ 1.2 and DAS28 ≤ 3.2; non-responders are all patients with ΔDAS28 ≤ 0.6 and those with ΔDAS28 > 0.6 but ≤ 1.2 and with DAS28 > 5.1; all the remaining patients are moderate responders. Generalized linear models for ΔDAS28 and logistic regression models for EULAR response criteria were fitted. Genotypes were considered according to an additive genetic model of minor allele counts (0, 1 or 2). Therefore positive regression coefficients indicate a better response associated with minor allele additive effects. Covariates included in the models were baseline DAS28, gender, the specific TNFi, and the Spanish or Greek origin. Statistica 7.0 (Statsoft, Tulsa OK) software was used to perform these analyses. Meta-analysis of the current study with all the available non-redundant results from previous studies was done. Specifically, information from 2466 patients included in previous studies and from 689 patients from the current study at 6 months was available corresponding to the three SNPs selected from Plant *et al*. [13]. The previous information was reported in [13] as stages 1 (WTCCC, n = 566), 2 (n = 379) and 3 (n = 341) from the UK; in [11] as the DANBIO register (n = 196); and in [12] as the stage 1 (combining two Dutch collections, n = 984). In turn, there were data from 3155 non-redundant patients corresponding to the eight Umicevic Mirkov *et al*. [12] SNPs. This information was available in a previous study [12] as four sample collections: stage 1 (jointly including Dream and ApotheekZorg, n = 882), stage 2 (REF collection, n = 954), WTCCC (n = 595) and ReAct (n = 272), and in the current study as 452 patients assessed at 3 months of follow-up. Finally, there was information for rs6427528 from 1178 patients treated with etanercept and assessed at any time from 3 to 12 months. They were reported in the current (n = 155) and previous studies (n = 1023) [14]. Previous collections were: REF (n = 365), BRAGGSS (n = 259), DREAM (n = 109), Portuguese (139), Kyoto (n = 88) and IORRA (n = 63). The reader is referred to the original manuscripts for the detailed definition of each sample collection. In addition, it should be noted that the number of patients included in the different studies, at different times or for different SNPs was variable. For example, the patients identified as WTCCC were not exactly the same in [13] and in [12], two studies that were evaluated at different times. Also, the number of samples used in [12] to replicate the SNPs from Plant *et al*. [13] was slightly different than the used to discover new associations. All these patient sets were combined with the fixed effects model of meta-analysis, weighting each cohort by the inverse variance method, except for SNPs showing significant heterogeneity (I^2^ > 50%). In this latter case, the random effects model according to DerSimonian and Laird was applied. These analyses were conducted with the R *metafor* package [31]. Post-hoc power analysis was conducted with G*Power 3 considering the response to treatment at any time [32].

## Results

The 755 patients with RA showed characteristics of a severe disease before starting treatment with TNFi (Table 1). This was indicated by frequent erosive arthritis (70.7%), high disease activity (mean DAS28 = 5.8) and moderate to severe disability (mean HAQ = 1.5). The patients presented this level of severity at the baseline in spite of previous treatment with a mean of 2.64 different conventional DMARDs. None of these previous drugs included a bDMARD as this was one of the exclusion criteria in our project. The patients received any of the three most common TNFi during the period of assessment, infliximab (52.6%), etanercept (20.5%) or adalimumab (26.9%). The TNFi were given in parallel with a conventional DMARD in 95.0% of the patients. However, treatment was inefficient in some patients, with a percentage of non-responders according to the EULAR criteria that remained fairly constant at the three evaluation times: between 18.1 and 20.8% (see Supporting information S3 Table for the drug-stratified data).

In our attempt to validate biomarkers of response to TNFi, we analyzed the 12 SNPs from GWAS in Table 2. Their genotypes passed quality control criteria, including call rate (> 99%), reproducibility (100%), fit to HWE (*P* > 0.05), and consistency with HapMap frequencies (all pair-wise comparisons P > 0.05). Unfortunately, linear regression did not show any significant association between the 12 SNPs and the predefined main outcome of response, ΔDAS28 at 6 months (Table 3), or with the secondary outcomes, ΔDAS28 at 3 or at 12 months (Table 3). Similarly, logistic regression did not show association of the SNPs with the non-responder *vs*. responder classification of the patients (S4 Table). In addition, the *CD84* SNP rs6427528, which was specifically associated with the response to etanercept in a previous GWAS [14], was not associated in this subset of our patients (B = 0.16, 95% CI -0.43, 0.76, *p* = 0.6 for ΔDAS28 at 3 months; and OR = 1.16, 95% CI (0.68–1.97), *p* = 0.6 for the responder vs. non-responder comparison; and similar results at 6 or 12 months).

**Table 3:** Results of the linear regression of SNP genotypes on the ΔDAS28 at the indicated times under TNFi treatment.

SNP	Mi/Ma^1^	3 mo.			6 mo.			12 mo.		
		B	SE	p	B	SE	p	B	SE	p
rs12142623	T/G	0.23	0.13	0.07	0.11	0.1	0.3	- 0.17	0.13	0.2
rs1568885	T/A	0.06	0.12	0.6	0.03	0.1	0.7	0.10	0.12	0.4
rs2378945	A/G	- 0.14	0.09	0.10	0.05	0.07	0.5	- 0.10	0.09	0.2
rs4651370	A/T	0.14	0.12	0.3	0.05	0.1	0.6	- 0.19	0.13	0.14
rs1447722	C/G	- 0.05	0.09	0.5	- 0.04	0.07	0.5	0.08	0.09	0.4
rs1813443	G/C	0.04	0.10	0.7	- 0.05	0.07	0.5	- 0.05	0.09	0.6
rs4411591	A/G	0.01	0.12	0.9	0.02	0.09	0.8	0.07	0.11	0.6
rs7767069	T/A	- 0.06	0.10	0.6	<0.01	0.08	1.0	- 0.08	0.09	0.4
rs7962316	G/A	0.09	0.09	0.4	0.06	0.07	0.4	- 0.01	0.09	0.9
rs1350948	A/G	<0.01	0.11	1.0	0.042	0.09	0.6	0.01	0.11	0.9
rs4694890	A/C	- 0.05	0.08	0.6	0.07	0.07	0.3	- 0.01	0.08	0.9
rs6427528	A/G	<0.01	0.13	1.0	- 0.04	0.11	0.7	0.01	0.13	0.9

^1^ Minor/major alleles. B = regression coefficient and SE, its standard error.

**Fig 1:**
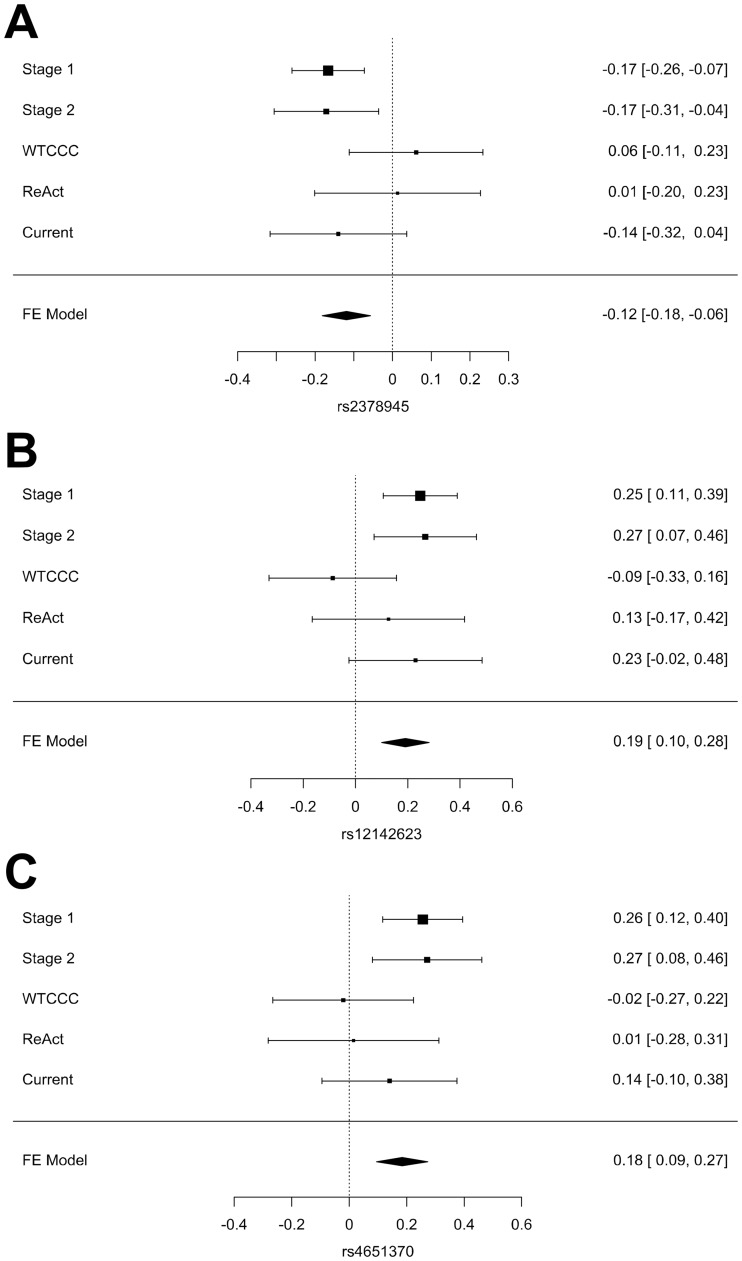
Meta-analysis of the three SNPs showing reinforced association with the current study. The coefficients of the regression with their 95% confidence intervals from each cohort and their fixed effects summary are presented. The forest plots correspond to A) rs2378945, B) rs12142623 and C) rs4651370. The Stage 1, Stage 2, WTCCC and ReAct data for these SNPs were exclusively reported in Umicevic Mirkov *et al*. [12].

Considering the convenience of summarizing the results, we performed post-hoc power analysis of our results, and combined meta-analysis with the previous data. The power analysis showed our study had only enough post-hoc power (1-β > 80%) to reproduce the rs4694890 and rs7767069 reported associations, with rs1350948 very near this level (77%). For the remaining SNPs, power was insufficient and assessment of their status relies more heavily in the combined meta-analysis than in the replication. The meta-analysis (Table 4) included a total of 3155 patients with information for the 3 SNPs identified by Plant *et al*. [13], and the same number, but not exactly the same patients, for the 8 SNPs drawn from Umicevic Mirkov *et al*. [12]. In turn, there were a total of 1178 patients treated with etanercept with information for the rs6427528 SNP identified by Cui *et al*. [14]. Three of the 12 associations were slightly reinforced by the meta-analysis including our results (Fig 1). The association of rs2378945 changed from *p* = 6.9 x10^-4^ to 1.8 x10^-4^; the corresponding to rs12142623 passed from *p* = 2.0 x10^-4^ to 4.2 x10^-5^; and the change for rs4651370 was from *p* = 1.1 x10^-4^ to 5.6 x 10^−5^. In contrast, four other SNPs were no longer associated with response to treatment and the remaining five SNPs showed decreased associations (Table 4).

**Table 4:** Summary statistics of the meta-analysis combining the current and previous studies.

SNP	Coefficient	CI	p	I^2^
rs7962316	0.07^a^	-0.07, 0.20	0.3	69
rs1350948	0.12^a^	-0.02, 0.25	0.09	58
rs4694890	-0.03^a^	-0.16, 0.10	0.6	74
rs2378945	-0.12	-0.18, -0.06	0.0002	45
rs12142623	0.19	0.10, 0.28	0.00004	38
rs1568885	0.17	0.08, 0.26	0.0002	0
rs1447722	0.07^a^	-0.05, 0.19	0.2	64
rs1813443	-0.12	-0.19, -0.05	0.0007	21
rs4651370	0.18	0.10, 0.27	0.00006	32
rs4411591	0.17	0.08, 0.26	0.0002	0
rs7767069	-0.13	-0.20, -0.07	0.0001	38
rs6427528^b^	0.33^a^	0.03, 0.64	0.03	66

^a^ Random effects meta-analysis was used because of significant heterogeneity

^b^ Only patients treated with etanercept, n = 1178

**Fig 2:**
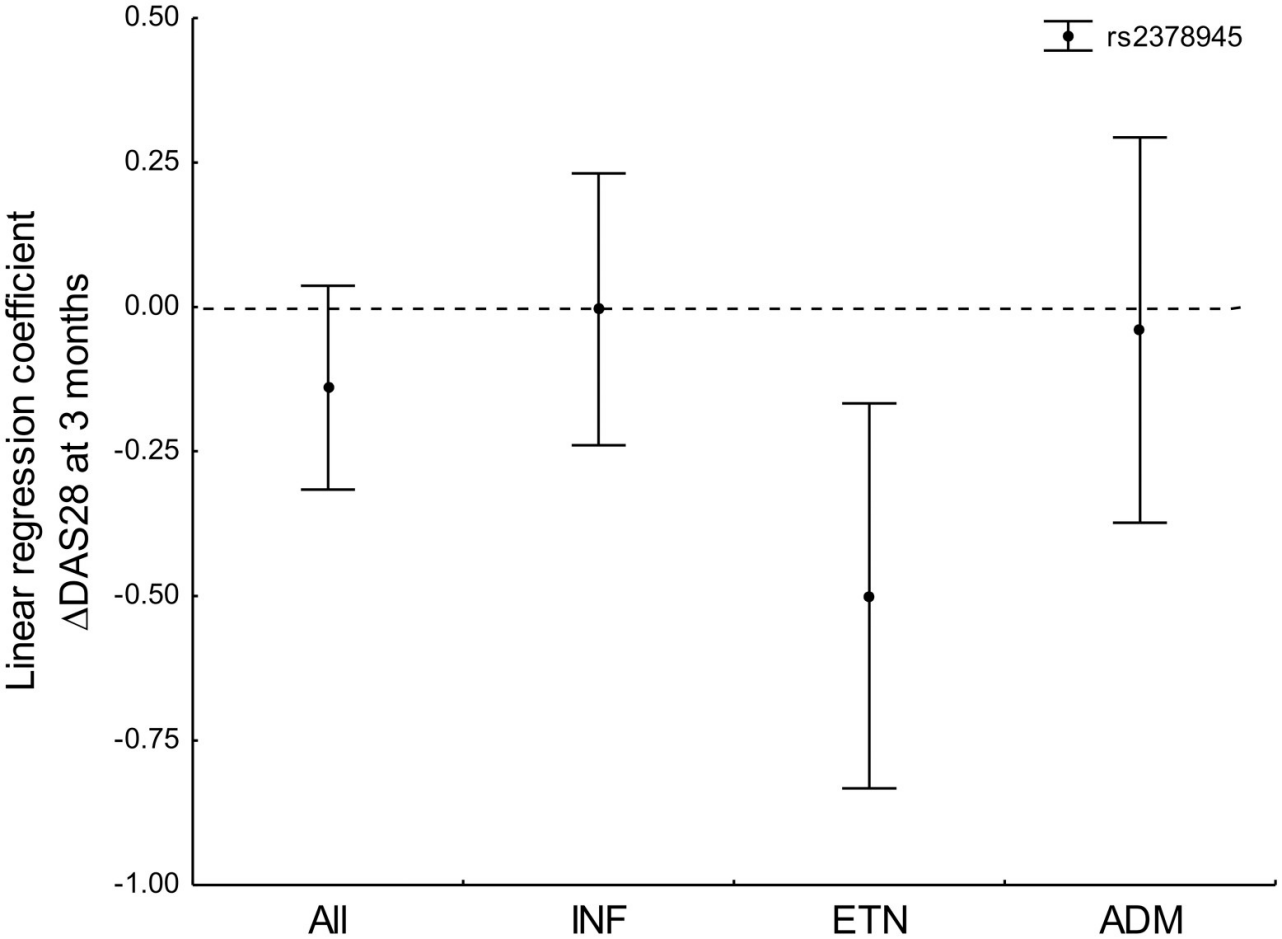
Representation of the linear regression coefficients and their 95% CI corresponding to rs2378945 genotypes in the multiple regression of ΔDAS28 at 3 months. The coefficients for the whole set of patients with RA (ALL) and in the subsets treated with each of the three TNFi (IFN = infliximab, ETN = etanercept, ADM = adalimumab) are shown.

To complete our study, we included an exploratory analysis stratified by the different TNFi. In this exploration, we uncovered association of the *NUBPL* rs2378945 SNP at 3 months with ΔDAS28 only in the etanercept-treated patients (Fig 2, B = -0.50, 95% CI (-0.82, -0.17), *p* = 0.003). This result represented less decrease of DAS28 in the patients bearing the minor allele of rs2378945. The association with reduced response was also reflected in the analysis of the EULAR response. In effect, the minor allele of rs2378945 was associated with less good responders compared with moderate responders and non-responders considered together (OR = 0.54, 95% CI = 0.31–0.96, *p* = 0.035), without significant distinction between moderate responders and non-responders (*p* = 0.7). The circumscribed association to the 3-month evaluation could be due to the progressive improvement observed in the patients treated with etanercept (non-responders changed from 20.9% at 3 months to 8.3% and 7.2% at 6 and 12 months, respectively). An improvement that was not observed in patients treated with the other TNFi (S3 Table).

## Discussion

None of the 12 GWAS-drawn SNPs showed association with the response to TNFi in our RA patients. This lack of replication is important because the SNPs were from high-quality studies and this is the first attempt of independent replication for 9 of them. Nevertheless, the combination of our results with the previous studies discriminated between 3 SNPs that showed strengthened association and the remaining 9 SNPs whose status was weakened without question. In any case, the low level of reproducibility reinforces the need for circumspect consideration and calls for more powerful studies. In this regard, the association of *NUBPL* rs2378945 with the specific response to etanercept could reflect the phenotype complexity.

Two of the three associations that were reinforced by our results could be considered as only one because rs12142623 and rs4651370 are near and in high linkage disequilibrium between them (r^2^ = 0.90 in subjects of European descent according to the Phase 3 of the 1000 Genomes project; https://ldlink.nci.nih.gov/). They could modify the expression of the neighboring *PLA2G4A* gene, which codes for the arachidonyl specific group IVA cytosolic phospholipase 2 enzyme. This enzyme regulates TNF-induced joint damaging mediators [33]. The other reinforced association corresponds to rs2378945, which is the same SNP we found associated with reduced response to etanercept in the drug-stratified analysis. This SNP is associated with the expression of the nearby *NUBPL* gene (GTEx, http://www.gtexportal.org.), which is a member of the ATP-binding protein family required for the assembly of the mitochondrial complex I. However, it has not any known relationship with inflammation or autoimmunity.

Three of the four SNPs that were no longer associated after meta-analysis (rs7962316, rs1350948, rs4694890) had a weak standing as biomarkers of response to TNFi even before our study. They were associated in the two first phases, but not in the third, of the original GWAS highlighting them [13]. Afterward, their status was further undermined by the lack of replication in two subsequent GWAS (although the time of assessment was different) [11,12]. Therefore, our results regarding these 3 SNPs could be taken as a confirmation of their lack of association with response to TNFi. Also, the 8 SNPs drawn from the Umicevic Mirkov *et al*. [12] report had been associated with response to TNFi in the first two phases of the original GWAS, but not in the additional replication collections from the same study [12]. The particularity with these 8 SNPs is that no other work had addressed them until now. Therefore, our study is the first independent validation and it differentiated between the three SNPs that were reinforced and the five SNPs that were more clearly questioned. Lastly, rs6427528 showed the strongest standing as biomarker before our study. It was associated with response to etanercept with a p-value that approached the GWAS cut-off [14]. However, not all the evidence in the original study was favorable to the association, and the meta-analysis of all results has shown a very weakened association.

The lack of reproducibility we have observed adds to the circumspection advocated in other analyses and reviews [6,23]. Currently, even the most promising biomarkers are not without doubts. For example, the first SNP that reached the GWAS level of significance, rs3794271 [34], was not replicated in a subsequent and larger study [35]. Other example is the mentioned rs6427528 in *CD84* [14] that has been very undermined in our meta-analysis. However, there are reasons to expect that some SNPs will be confirmed in future studies. They could be among the SNPs that have demonstrated significant association in a meta-analysis [6], or the associations below the 5 x10^-8^ threshold from two recent GWAS [18,19].

The lack of reproducibility cannot be attributed to a weak genetic component in the response to TNFi phenotype. On the contrary, the heritability of ΔDAS28 has been estimated at 0.59–0.71, which similar to other complex traits [36]. Nonetheless, the genetic structure of this component seems very complex [23,37], resembling other common diseases and biological traits as exemplified by the more than one hundred RA susceptibility loci [38]. This polygenic causality means that most loci have a small effect, requiring larger studies for their identification than the available for response to TNFi.

Other aspects that could contribute to the lack of reproducibility include variability of the response along the time and in function of the drug. The effect of time is well-known in clinical practice, where primary and secondary failure to TNFi are distinguished [39]. In this regard, there are some SNPs showing association with the response only at a given time of assessment [6], as the observed in the current study with *NUBL*. The effect of a specific drug, in turn, could be justified by the known differences between the TNFi [25,26]. Its potential impact on the identification of biomarkers has been revealed by the higher heritability of the response to the monoclonal antibodies (infliximab and adalimumab) than to etanercept (a soluble receptor) [23] and several genetic studies leading to the identification of drug-specific associations [14,19,21,22]. The specific association of the *NUBPL* SNP with the response to etanercept we have found contributes to the accumulating evidence in this direction.

Another factor likely contributing to the lack of reproducibility is the heterogeneity of the outcomes [36,40]. This issue has been poorly studied, but there are differences in heritability between the components of the DAS28 [36]. The highest estimates of heritability were obtained for the change in the swollen joint count (ΔSJC) and in the tender joint count (ΔTJC). They were followed by ΔDAS28, while heritability of change in the erythrocyte sedimentation rate (ΔESR) and in the general health scale (ΔVAS) were negligible [36]. Therefore, future studies could take advantage of this information focusing on the high heritability components. There are also reasons to prefer ΔDAS28 over the EULAR response. As a general rule, the dichotomized variables have many disadvantages in comparison with the continuous ones [41,42]. In consequence, ΔDAS28 should be more informative and less prone to false positive results than the EULAR response, but no study has compared their performance for the search of biomarkers. Finally, a large within-patient variation at successive visits has been described [40]. This variability decreased the apparent heritability of the response and reduced the power to identify genetic biomarkers. Consequently, the use of the mean of the outcomes assessed at short time intervals has been recommended [40].

Our study serves as an invitation to revise the search for genetic biomarkers of response to TNFi given the difficulties encountered for their validations. It also serves to highlight the complexity of the response phenotype, which likely involves many loci with small effects, and heterogeneity in function of the specific drug and time of assessment. Therefore, identification of reproducible biomarkers will require larger collections of samples and more aggressive patient stratification. In addition, this identification would benefit from the incorporation of alternative and frequent measures of response.

## Supporting information

S1 Table Raw individual data of the patients included in the study. (XLSX)

S2 Table Primers and probes used for genotyping the studied SNPs. (XLSX)

S3 Table Response to the different TNFi according to the EULAR criteria. The percentage, %, and the number, (), of patients in each stratum are provided.
(XLSX)

S4 Table Results of the logistic regression of SNP genotypes on the EULAR criteria at the indicated times under TNFi treatment. The two compared groups were responders (good responder + moderate responders) and non-responders.
(XLSX)
